# Trends in Specialty Training and National Institutes of Health Funding Among Surgeon-Scientists

**DOI:** 10.1097/AS9.0000000000000521

**Published:** 2024-11-25

**Authors:** Gabriel Velez, Vinit B. Mahajan, Ronald J. Weigel, Steven R. Lentz

**Affiliations:** From the * Medical Scientist Training Program, University of Iowa, Iowa City, IA; † Department of Ophthalmology, Molecular Surgery Laboratory, Stanford University, Palo Alto, CA; ‡ Department of Ophthalmology, Byers Eye Institute, Stanford University, Palo Alto, CA; § Veterans Affairs Palo Alto Health Care System, Stanford University, Palo Alto, CA; ‖ Department of Surgery, University of Iowa, Iowa City, IA; ¶ Department of Internal Medicine, University of Iowa, Iowa City, IA.

**Keywords:** academic surgery, funding, grants, K-award, National Institutes of Health, surgeon-scientist

## Abstract

**Purpose:**

To determine if dual-degree training [ie, completion of a National Institutes of Health (NIH)-funded MD/PhD program], among other professional development and demographic variables, predicted academic productivity (eg, K-to-R conversion, number of publications, etc.) among early-career surgeon-scientists.

**Methods:**

We analyzed publicly available data from the National MD/PhD Program Outcomes Study and the Association of American Medical Colleges Graduate Medical Education Track database to identify trends in the number and proportion of MD/PhD graduates pursuing surgical specialties. NIH Research Portfolio Online Reporting Tool Expenditures and Results was interrogated to identify a cohort of early-career academic surgeon-scientists receiving K-awards from 2011 to 2021.

**Results:**

The total number of MD/PhD program graduates completing Graduate Medical Education training increased each decade after the Medical Scientist Training Program was established by the National Institute of General Medical Sciences, but the proportion completing surgical specialties did not change significantly (*P* = 0.96) from 1965 to 2014. More recent residency match trends demonstrate an increase in both the proportion and number of MD/PhD graduates entering surgical specialties, with 21.5% entering surgical residency training in 2020. Among 476 early-career academic surgeon-scientists receiving K-awards at 70 institutions, 27% were faculty members at only 4 universities, suggesting that federally funded surgeon-scientists are concentrated at a small number of institutions. Although MD/PhD graduates represented only 2.3% of active surgical residents from 2011 to 2020, they constituted a much higher fraction of K-awardees (29%). Of 296 surgeon-scientists who completed K-awards, 35% successfully obtained an R01-equivalent award.

**Conclusions:**

These findings emphasize the need for comprehensive career development and institutional resources to support early-career surgeon-scientists.

## INTRODUCTION

Surgically treatable conditions are estimated to account for one-third of the global disease burden.^1^ Surgeons who are rigorously trained in basic science and translational research possess the ability to leverage their clinical and scientific expertise to uncover biological mechanisms underlying surgical diseases, access human tissue samples, and craft innovative solutions for surgical patients. Surgeon-scientists have been instrumental in advancing their respective fields; the Nobel Prize in Physiology or Medicine has been awarded to 9 surgeons.^2^ Pioneering surgeon-scientists include Thomas E. Starzl for his contributions to solid organ transplantation, Werner Forssmann for performing the first cardiac catheterization, and James Bainbridge for performing the world’s first gene replacement surgery in the eye.^3^ Despite these achievements, National Institutes of Health (NIH) funding to surgical departments declined by 27% between 2007 and 2014.^4^ Surgeons in the United States submit 40% fewer grant proposals than their nonsurgical colleagues and are less likely to receive funding when they do apply (16% success rate compared to 20% for nonsurgeons).^5–7^ From 2010 to 2020, the proportion of NIH-funded physicians who were surgeons rose from 5% to 9%,^8^ but surgeons remain underrepresented among NIH-funded physicians (surgeons make up 18% of the physician workforce).^7,8^

Barriers to establishing a successful career as a surgeon-scientist are related to the lack of NIH support for research training during surgical residency, the changing academic medical environment, including increased pressure for clinical productivity, increasing complexity of both the medical regulatory system and scientific research, and competition with nonclinician scientists for limited research funding.^5^ The decline in the proportion of basic and translational science abstracts (48–27%) submitted to the Academic Surgical Conference from 2011 to 2015 suggests that many academic surgeons are shifting their research focus towards health services research (HSR).^7,9^ The COVID-19 pandemic has further threatened surgeon-scientist career development by reducing research time to prioritize COVID-19-related clinical efforts.^10,11^

There are numerous training pathways for aspiring surgeon-scientists with entry points at the undergraduate, medical school, and postgraduate training levels. Formal research training can be obtained during medical school through dual-degree training (eg, MD/PhD programs) or surgical residencies that offer dedicated time for research training, often with the option of a masters or doctoral degree.^12^ MD/PhD programs offer rigorous structured research training and scientific career development experiences, both of which are critical for the development of a career in academic surgery. However, surgeon-scientists are often underrepresented as mentors for MD/PhD students, and some medical school faculty (surgical and nonsurgical faculty alike) may hold the perception that surgeons cannot be successful scientists in today’s environment, which may dissuade MD/PhD students from entering surgical specialties.^13^ Even when MD/PhD graduates enter surgical residencies, the “research gap” that occurs between their terminal research experience and first faculty position can adversely affect their readiness during the critical early years of their academic surgery career.

Previous studies analyzing trends and demographics in NIH early-career surgeon-scientist grant recipients have included only awardees appointed in departments of surgery in the NIH Research Portfolio Online Reporting Tool Expenditures and Results (RePORTER) database.^14^ This approach fails to capture the complete spectrum of surgical subspecialties, thereby underrepresenting the number of NIH grants attributable to surgeons. In the current study, we analyzed a cohort of 476 early-career academic surgeons across all surgical subspecialties receiving NIH K-awards from 2011 to 2021. Additionally, we studied the number and proportion of MD/PhD graduates completing Accreditation Council for Graduate Medical Education-accredited graduate medical education (GME) programs in surgical specialties in the last five decades and compared these results to those of nonsurgical specialties.

## METHODS

### Study Approval

The study was approved by the University of Iowa Institutional Review Board (IRB) (protocol #202202108) and adhered to the tenets set forth in the Declaration of Helsinki.

### Database Interrogation

Publicly-available data from the National MD/PhD Program Outcomes Study were analyzed. As described in the original report,^15^ data were collected from surveys distributed to 10,591 MD/PhD program graduates. Briefly, survey data from 6786 MD/PhD program graduates (64.1% response rate) representing 80 programs, including 44 NIH-funded Medical Scientist Training Programs, were collected. Of the 6786 respondents, 4759 had completed postgraduate residency training. The survey was approved by the Association of American Medical Colleges (AAMC) IRB. Data from selected survey questions were interrogated to determine trends in MD/PhD program graduates who had completed postgraduate clinical training up to the 2014 academic year. Additionally, the GME Track and Student Records System databases from the AAMC were interrogated to identify the number of US MD/PhD dual-degree program graduates entering residency programs from 2011 to 2020, by specialty. The proportion of MD/PhD graduates pursuing a particular specialty was determined by dividing the number of MD/PhD graduates in that specialty by the total number of unique MD/PhD graduates in the respective year. The representation of MD/PhD graduates in a particular specialty was calculated by dividing the number of MD/PhD graduates by the total number of active residents in that specialty in the given year. Linear regression analysis was used to determine whether the proportion or representation of MD/PhD graduates in certain specialties had changed significantly over time. Data were analyzed using GraphPad Prism 9.

### Cohort Analysis

A list of NIH early-career development grants awarded to faculty members appointed to surgical departments (ie, bariatric, breast, cardiothoracic, colorectal, endocrine, general surgery, neurosurgery, obstetrics and gynecology, ophthalmology, otolaryngology, orthopedics, pediatric, plastic, surgical oncology, transplant, trauma, urology, or vascular surgery), ranging from the fiscal year 2011 to 2021, was curated using the NIH RePORTER tool. A total of 910 NIH K-awards to faculty members in surgical departments were found. Institutional grants (eg, K12, K18, and K24) were excluded. The project description for each award was reviewed and categorized into a single project type that best fits the definition. Basic science projects were defined as any study utilizing benchwork in a laboratory. Translational research was designated as a study that applied findings from basic science to methods that address medical needs. HSR was specified as a study that addressed access to healthcare, healthcare costs, or patient outcomes. Faculty listings from institutional websites and publicly-available CVs for all 910 K-awardess were reviewed to identify 476 active early-career surgeon-scientists (with active clinical offices and ongoing research participation) from this list. Additional information (eg, subspecialty, advanced degrees, etc.) also was obtained from this review. PubMed was interrogated to obtain publication data.

## RESULTS

### MD/PhD Program Graduates are Underrepresented in Surgical Specialties

**FIGURE 1.**
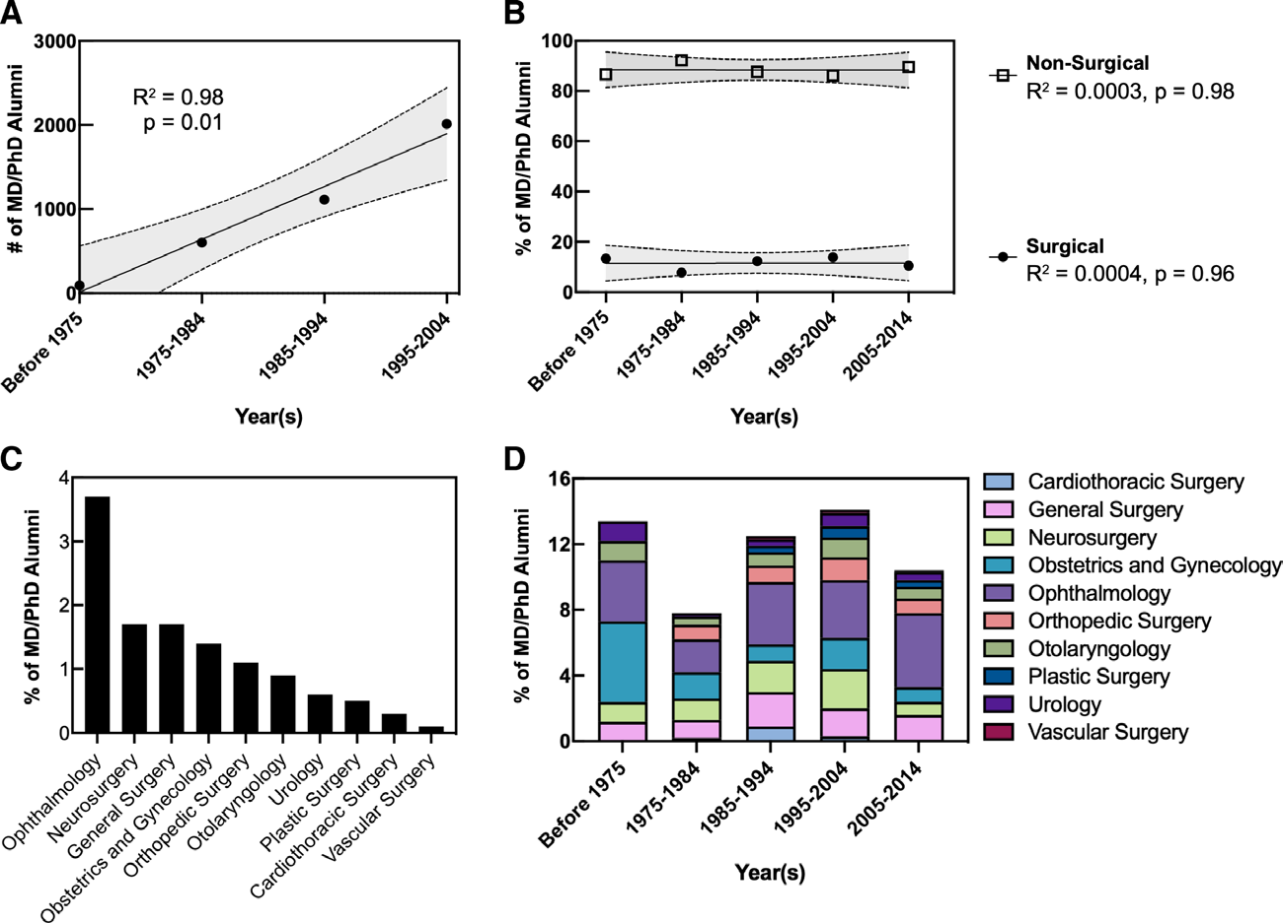
Trends in MD/PhD program graduates who completed graduate medical training in surgical specialties by 2014. A, Number of MD/PhD program graduates that completed graduate medical education (GME) each decade since the advent of the NIH-funded MSTPs in 1965. The total number of MD/PhD alumni completing GME has increased over time (R^2^ = 0.98 *P* = 0.01). The 95% confidence interval is denoted by the dashed, shaded region. B, Percentage of MD/PhD program graduates completing GME training in nonsurgical and surgical specialties. The proportion of MD/PhD program graduates completing nonsurgical (R^2^ = 0.0003; *P* = 0.98) versus surgical (R^2^ = 0.0004; *P* = 0.96) residencies has not changed significantly over this period. C, Percentage of total MD/PhD program graduates from 1975 to 2014 who completed GME training in surgical specialties sorted by subspecialty. D, Percentage of total MD/PhD program graduates from each decade completing GME training in surgical specialties sorted by surgical subspecialty. Data were adapted from the National MD/PhD Program Outcomes Study.

We interrogated data from the National MD/PhD Program Outcomes Study to identify historical trends among US MD/PhD program graduates who had completed GME training by 2014 (Fig. 1). The number of MD/PhD program graduates completing GME training increased during each decade after the launch of NIH-funded Medical Scientist Training Programs in 1965 (R^2^ = 0.98; *P* = 0.01; Fig. 1A). We then grouped MD/PhD program graduates based on their GME specialty selection (ie, surgical vs nonsurgical). For this analysis, the following specialties were considered surgical in nature: general surgery, cardiothoracic surgery, plastic surgery, colorectal surgery, vascular surgery, neurosurgery, obstetrics and gynecology, ophthalmology, otolaryngology, orthopedics, and urology. The mean proportion of MD/PhD program alumni completing surgical residency training from 1965 to 2014 was 11.6 ± 2.5%; this proportion did not change significantly over this period (R^2^ = 0.0004; *P* = 0.96; Fig. 1B). The gender gap was similar for surgical specialties (28% female) compared with nonsurgical specialties (32% female). Among the 4759 MD/PhD alumni who completed postgraduate clinical training, the most represented surgical subspecialties were ophthalmology (n = 174; 3.7%), neurosurgery (n = 83; 1.7%), general surgery (n = 79; 1.7%), and obstetrics and gynecology (n = 68; 1.4 %; Fig. 1C). The distribution of surgical subspecialty choice did not change significantly over time (Fig. 1D). These results indicate that the total number but not the proportion of MD/PhD alumni completing GME training in surgical specialties increased from 1965 to 2014.

**FIGURE 2.**
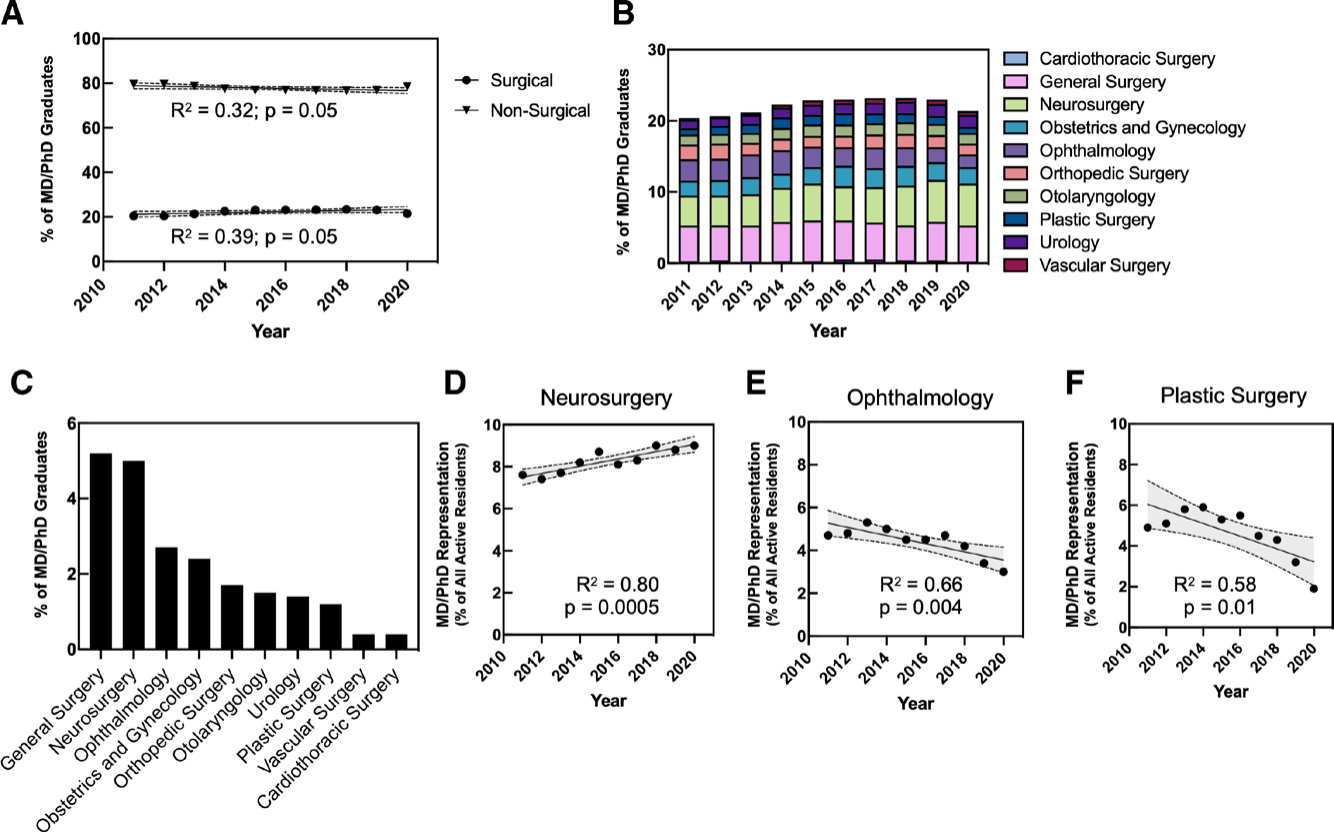
Trends in MD/PhD program graduates entering graduate medical training in surgical specialties from 2011 to 2020. The GME Track and SRS databases from the AAMC were interrogated to identify the number of US MD/PhD program graduates entering residency programs from 2011 to 2020, by specialty. A, Percentage of MD/PhD program graduates entering GME training in nonsurgical and surgical specialties from 2011 to 2020. The proportion of MD/PhD program graduates entering surgical residency programs has tended to increase during this period (R^2^ = 0.39; *P* = 0.05). B, Percentage of MD/PhD program graduates entering GME training in surgical specialties from 2011 to 2020, sorted by subspecialty. C, Mean percentage of MD/PhD graduates from 2011 to 2020 entering GME training in surgical specialties. D–F, Representation of MD/PhD program graduates (% of all residents who are MD/PhD graduates) entering GME training in neurosurgery, ophthalmology, and plastic surgery over time. There was a significant increase in representation of MD/PhD program graduates among neurosurgery residents (R^2^ = 0.80; *P* = 0.0005) (D) and significant decreases in representation of MD/PhD program graduates among ophthalmology residents (R^2^ = 0.66; *P* = 0.004) (E) and plastic surgery residents (R^2^ = 0.58; *P* = 0.01) (F). The 95% confidence intervals are denoted by the dashed, shaded region.

We next interrogated residency databases and tracking systems (AAMC GME Track and Student Records System) to identify more recent GME specialty selection trends among MD/PhD program graduates (Fig. 2). This dataset includes demographic information on MD/PhD program graduates entering residency programs from 2011 to 2020 as opposed to the National MD/PhD Program Outcomes Study, which includes data on graduates who had completed postgraduate clinical training by 2014. During the decade from 2011 to 2020, over 20% of MD/PhD graduates entered surgical residency programs, and there was a modest increase in both the number and percentage of MD/PhD program graduates entering surgical residency programs, from 399 (20.5%) in 2011 to 448 (21.5%) in 2020 (R^2^ = 0.39; *P* = 0.05; Fig. 2A). The distribution of surgical subspecialty choice during this period is illustrated in Fig. 2B. The top 4 GME surgical specialties with the highest number of active MD/PhD program graduates from 2011 to 2020 were the same as during the previous 5 decades, although the proportions have shifted significantly between the 2 time periods: general surgery (n = 107; 5.2%), neurosurgery (n = 103; 5.0%), ophthalmology (n = 56; 2.7%), and obstetrics and gynecology (n = 50; 2.4%; Fig. 2C).

We next analyzed the representation of MD/PhD program graduates among all active trainees in US residency programs, by specialty. MD/PhD program graduates represented 3.4% of total active residents, and 2.3% of active residents in surgical training programs, between 2011 and 2020. The surgical specialty with the highest representation of MD/PhD graduates was neurosurgery (n = 103; 8.3%). The representation of MD/PhD graduates among neurosurgery residents increased significantly from 2011 to 2020 (R^2^ = 0.80; *P* = 0.0005; Fig. 2D). Conversely, the representation of MD/PhD graduates among ophthalmology and plastic surgery residents declined significantly in this period (R^2^ = 0.66; *P* = 0.004 and R^2^ = 0.58; *P* = 0.01, respectively; Fig. 2E,F). MD/PhD graduates were underrepresented in orthopedic surgery, obstetrics and gynecology, otolaryngology, and urology. Taken together, these data support a recent upward trend in the number and percentage of MD/PhD graduates entering residency training in surgical specialties.

### Trends and Characteristics of Early-Career Academic Surgeon-Scientists

**FIGURE 3.**
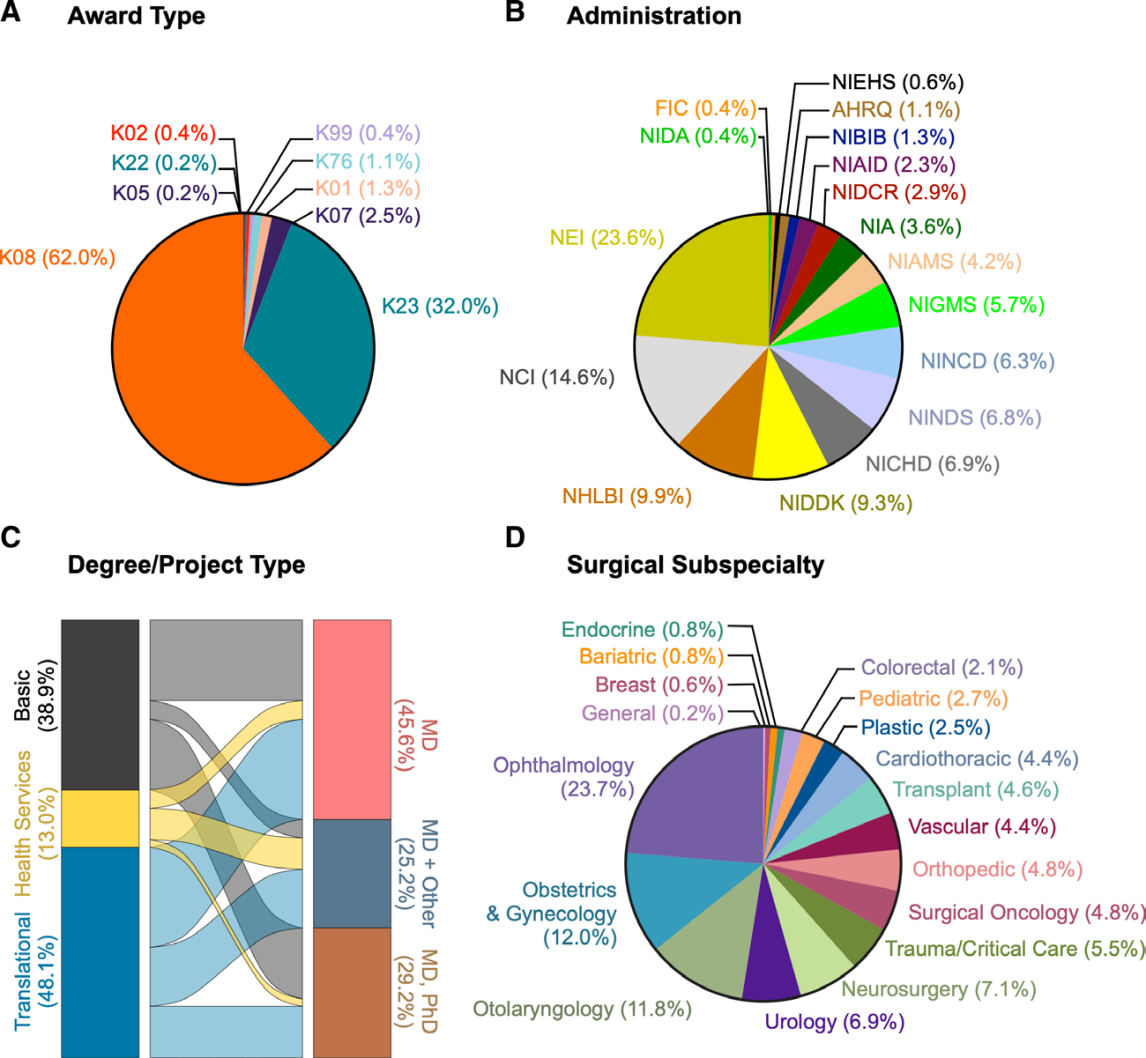
Trends of clinically active early-career surgeon-scientists. A total of 910 principal investigators with primary appointments in US surgical departments received an NIH K-award between January 1, 2011, and December 31, 2021. A total of 362 recipients were excluded for not having a medical degree, completing a nonsurgical residency, or were not clinically active. Institutional and mid-career level awards (eg, K12, K18, and K24; n = 72) were also excluded. A total of 476 recipients were included in the analysis. A, Distribution of K-awards among early-career surgeon-scientists sorted by award type. B, Distribution of K-awards among early-career surgeon-scientists sorted by NIH funding administration. C, Distribution of K-awards among early-career surgeon-scientists sorted by project type and academic degree held by the principal investigator. Results are represented as an alluvial plot (D) Distribution of K-awards among early-career surgeon-scientists sorted by the surgical subspecialty of the principal investigator.

To identify trends and characteristics of surgeon-scientists at the early-career level, we identified a cohort of clinically active early-career surgeon-scientists in the US using NIH RePORTER. A total of 910 NIH K-level grants were awarded to faculty members in surgical departments between January 1, 2011, and December 31, 2021. Three hundred and sixty-two awardees were excluded for not having a medical degree, practicing in a nonsurgical specialty, or not being clinically active. Seventy-two institutional and mid-career level awardees (eg, K12, K18, and K24) were also excluded, resulting in 476 K-awardees appointed at 70 universities (Supplemental Table S1, http://links.lww.com/AOSO/A436) who were included in the analysis. The cohort was 37% female (n = 176; Table 1). Notably, 27.1% (n = 129) of K-awards were awarded to faculty members at 4 universities (Supplemental Table S1, http://links.lww.com/AOSO/A436). K08 (n = 296, 62%) and K23 (n = 153, 32%) awards comprised over 90% of the NIH grant mechanisms (Fig. 3A). Nearly 50% of the grants were awarded by the National Eye Institute (n = 112, 23.6%), the National Cancer Institute (n = 69, 14.6%), or the National Heart, Lung, and Blood Institute (n = 47, 9.9%; Fig. 3B). Of the 476 K-awards, 185 (38.9%) were categorized as basic science, 229 (48.1%) were translational, and 62 (13.0%) were HSR. Two-hundred and seventeen (45.6%) awardees had an MD degree only, 139 (29.2%) had both an MD and PhD, and 120 (25.2%) had an MD and another advanced degree (eg, MPH, MBA, MS, etc.; Fig. 3C). Based on surgical subspecialty, the largest number of recipients were ophthalmologists (n = 113, 22.6%), followed by obstetricians/gynecologists (n = 57, 19.3%) otolaryngologists (n = 56, 10.4%), and neurosurgeons (n = 34, 6.6%; Fig. 3D). Among the 139 MD/PhD K-award recipients, a majority were ophthalmologists (n = 56, 40.3%), followed by otolaryngologists (n = 19, 13.7%), and neurosurgeons (n = 11, 7.9%; Supplemental Table S2, http://links.lww.com/AOSO/A436). The surgical subspecialties with the lowest representation among 476 K-award recipients were general surgery (n = 1, 0.2%), breast surgery (n = 3, 0.6%), endocrine surgery (n = 4, 0.8%), and bariatric surgery (n = 4, 0.8%). Among the 139 MD/PhD K-award recipients, the subspecialties with the lowest representation were breast surgery (n = 1, 0.7%), endocrine surgery (n = 1, 0.7%), and vascular surgery (n = 1, 0.7%; Supplemental Table S2, http://links.lww.com/AOSO/A436).

**TABLE 1.** Demographics of Clinically Active Surgeon-Scientists who Received K-Awards

	Surgeon K-Recipients	Completed K-Awards**	Active R01-Award(s)
Total	n = 476 (n%)	n = 296 (n%)	n = 104 (n%)
Sex			
Female	176 (37.0)	111 (37.5)	27 (26.0)
Male	300 (63.0)	185 (62.5)	73 (74.0)
Degree(s)			
MD*	217 (45.6)	164 (55.4)	47 (45.2)
MD, PhD	139 (29.2)	101 (34.1)	33 (31.7)
MD + Other†	120 (25.2)	31 (10.5)	24 (23.1)
Publications	Mean ± SD (Range)		
Total publications	86 ± 89 (1–547)	94 ± 98 (1–547)	105 ± 99 (2–547)
First author	17 ± 21 (0–195)	18 ± 24 (0–195)	19 ± 20 (0–193)
Senior author	30 ± 45 (0–238)	34 ± 49 (0–238)	40 ± 47 (0–238)

* Or equivalent degree (eg, DO, MBBS, MBChB, etc.).

† Other advanced degree (eg, MPH, MBA, etc.).

‡Project end date on or before 2022 fiscal year.

**FIGURE 4.**
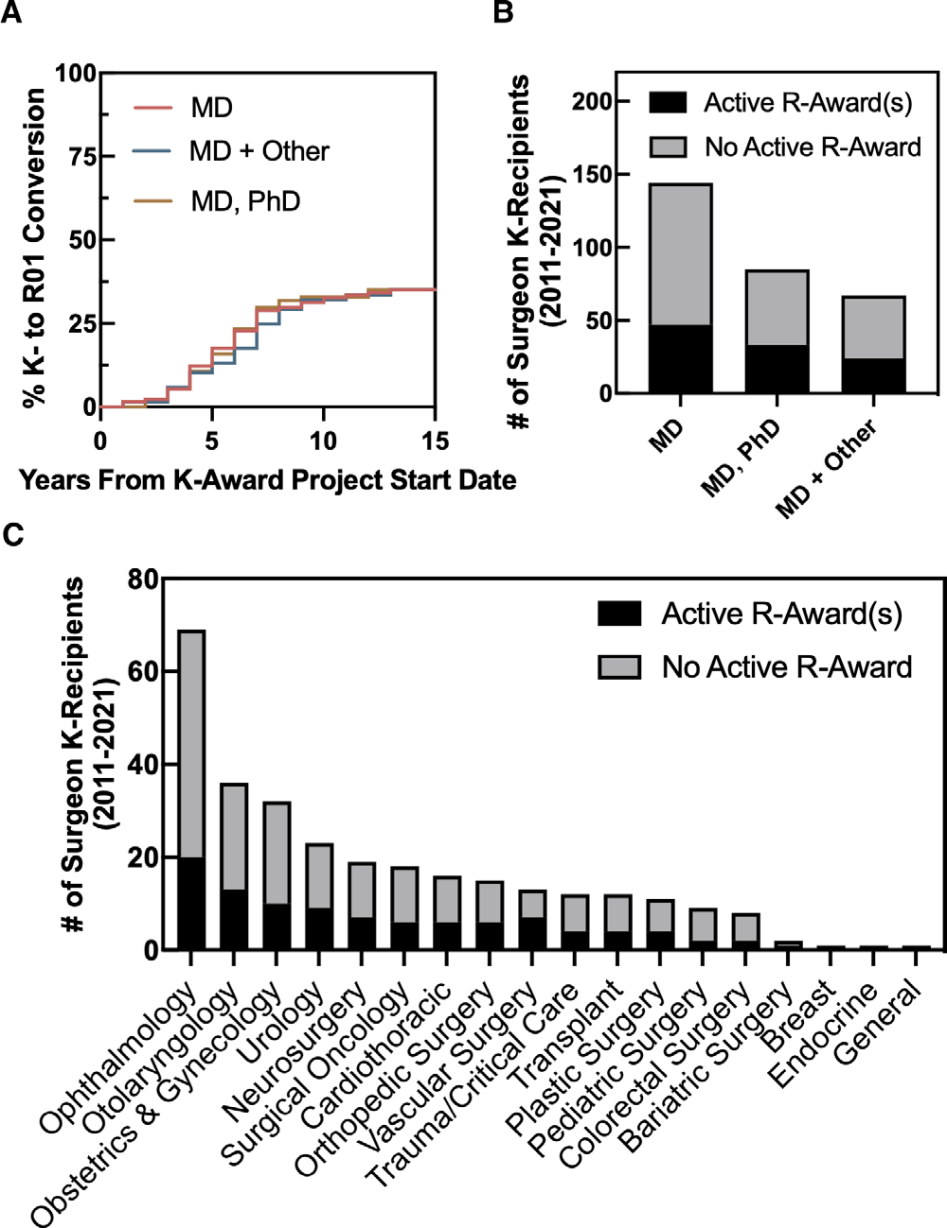
K-to-R conversion rates of clinically active surgeon-scientists. Of 296 surgeons who received K-awards with a project end date on or before the end of the 2021 fiscal year, 104 had received an active R01 (or equivalent) award as of 2022. A, Kaplan–Meyer analysis of the time to R01 conversion of surgeon K-awardees (n = 296). The median time from K-to-R01 conversion among successful converters (n = 104) was 6 years. B, Distribution of R01 awards of early-career surgeon-scientists sorted by degree type of the principal investigator. C, Distribution of R01 awards of early-career surgeon-scientists sorted by the surgical subspecialty of the principal investigator.

The conversion rate from K-award to R01-equivalent awards approximates the attrition rate among surgeon-scientists.^16^ Therefore, we calculated the K-to-R conversion rate for 296 K-award recipients with a project end date on or before the end of the 2021 fiscal year. Of these 296 K-award recipients, 104 (35.1%) held an active R01 (or equivalent) award as of 2022 (Table 1). The rate of K-to-R conversion was 24.3% for female K-awardees (ie, 27 out of 111 completed K-awards) and 39.5% for male K-awardees (ie, 73 out of 185). The rate of K-to-R conversion for the MD-only group was 29% (ie, 47 out of 164) versus 33% for the MD/PhD (ie, 33 out of 101) and 77% for the MD + other group (ie, 24 out of 31; Table 1). The median time from K-to-R conversion among successful converters in this cohort was 6 years, which is comparable to previously published K-to-R conversion times for physician-scientists in general (median K-to-R conversion time = 4.7 years; Fig. 4A).^17^ This trend did not change significantly when R01-awardees were grouped by academic degree or gender (MD degree only, 5.5 years; MD/PhD, 6 years; MD and another advanced degree, 6.5 years; Fig. 4A). Among the successful R01 converters, 27 (25.9%) were female, 47 (45.2%) had a MD degree only, 33 (31.7%) had both MD and PhD degrees, and 24 (23.1%) had an MD and another advanced degree (Fig. 4B and Table 1). Successful R01 converters had a significantly higher number of total and senior author publications compared with the entire cohort of K-recipients (*P* = 0.02 and *P* = 0.003, respectively; Table 1). Based on subspecialty, the largest number of R01 converters were ophthalmologists (n = 20, 19.2%), followed by otolaryngologists (n = 13, 12.5%), obstetricians/gynecologists (n = 10, 9.6%), and urologists (n = 9, 8.6%; Fig. 4C).

## DISCUSSION

The 2014 NIH Physician-Scientist Workforce Working Group report raised concerns about the small size and instability of the physician-scientist career development pipeline.^18^ Despite steady growth in the number of MD/PhD program graduates over the last 5 decades (Fig. 1A), physician-scientists still make up only a small percentage of US medical school graduates.^19^ The same is true for residency training programs. We found that MD/PhD program graduates comprised 3.4% of all active trainees, and only 2.3% of active surgical trainees, in US residency programs between 2011 and 2020. Our findings indicate that the proportion of MD/PhD program alumni completing surgical residency training did not change significantly from 1965 to 2014; however, more recent residency match trends do reflect increased interest in surgical specialty training among MD/PhD graduates during the last decade. The surgical residencies with the highest numbers of MD/PhD graduates are general surgery, neurosurgery, ophthalmology, and obstetrics and gynecology, although many other surgical specialties are represented.

A number of factors may explain why surgical residency programs historically have been less successful than nonsurgical programs in recruiting MD/PhD graduates, including a lack of exposure of MD/PhD trainees to surgeon-scientist mentors, underrepresentation of surgical specialties in medical school curricula, and a perceived lack of research opportunities in the surgical sciences.^4^ MD/PhD trainees typically have less time on the wards after completing their PhD training compared with their MD colleagues, which may leave little time for elective advanced surgical clerkships.

Although MD/PhD program graduates represented only 2.3% of active surgical residents, they constituted a much higher fraction of K- and R awardees in the early-career surgeon-scientist cohort (Table 1). Despite this, there was no significant difference between the rate of K-to-R conversion for MDs versus MD/PhDs (29% and 33%, respectively). While PhD training may improve the readiness of early-career surgeon-scientists during critical transition points, our results suggest that it is not a prerequisite to success or productivity during the early years of their academic surgery career. Rather, the choice of whether or not to pursue PhD training should be weighed with the individual research interests of the prospective surgeon-scientist (eg, basic vs translational vs HSR). Interestingly, 27% of the K-awards were awarded to surgeon-scientists at only 4 institutions (Supplemental Table S1, http://links.lww.com/AOSO/A436). This concentration of NIH-funded surgeon-scientists at a relatively small number of academic institutions suggests that the scope of infrastructure and support available to early-career surgeon-scientists may vary significantly between surgical departments. An alternative explanation for this observation could be that the observed concentration of NIH-funded surgeon-scientists at a relatively small number of institutions reflects the composition of the faculty in those programs (eg, the size of the department and/or number of faculty members who are active researchers in the department). Such information regarding the composition of faculty at respective institutions is not easily ascertained from our NIH RePORTER dataset. However, these are important considerations when discussing the scope of infrastructure and support available to early-career surgeon-scientists at various institutions.

The career development pathway for surgeon-scientists is more variable than for nonsurgeon-scientists and poses unique challenges in today’s academic medical environment. Survey-based studies have identified factors contributing to success among early-career surgeon-scientists, including both environmental (ie, mentorship, protected research time, financial support, and departmental culture) and individual factors (ie, perseverance, intellectual drive, and work/family balance).^14^ Among the identified barriers to success were departmental factors (ie, lack of mentorship, funding, and support of leadership), increased clinical demands, financial factors (ie, lower salary), cultural/social issues (eg, racism and sexism/gender bias), and the need for active surgical practice to maintain technical expertise.^14^ There is concern that surgeon-scientists are falling behind in basic science research and are disproportionately affected by declining NIH funding rates compared with other medical disciplines.^8,20^ Structured training and deliberate recruitment of early-stage surgeon-scientists are required to reinforce the surgeon-scientist pipeline. Formal scientific training, either in the form of dual-degree training or dedicated research training blocks during surgical residency must be coupled with early engagement of trainees by inspirational mentors and comprehensive surgeon-scientist career development programs to reduce attrition rates at key transition points (eg, between the terminal research experience and first faculty appointment).^21^

Our study has several limitations that are inherent to its retrospective design, particularly the use of multiple datasets and incomplete survey response rates. For example, the National MD/PhD Program Outcomes Study reported a survey response rate of 64.1%, which raises the possibility that response bias may have impacted the data. Additionally, this survey focused only on MD/PhD program alumni and did not collect data from physician-scientists without a PhD degree. As highlighted in our cohort of early-career surgeon-scientists, a great deal of surgical research, both basic and clinical, is currently being performed by non-MD/PhD graduates. The NIH RePORTER dataset is limited to NIH-funded surgeon-scientists. While K-to-R conversion is a metric of obvious import, we did not analyze early-career surgeons who received research funding from other organizations (eg, Department of Defense or Veteran’s Affairs). Thus, our analysis potentially excludes a significant number of academically successful surgeon-scientists who may not have applied for K-awards. Our analysis of this dataset was based on surgical subspecialty rather than department. We recognize that departmental structures differ by institution (eg, cardiothoracic surgery could be a division within a department of surgery or a separate department) and some specialty categories may be integrated (e.g., plastic and vascular surgery). Finally, the relatively small sample size of surgeon-scientists in certain subspecialties limited our ability to identify meaningful trends. Despite these limitations, our findings emphasize the need for comprehensive career development and institutional resources to support early-career surgeon-scientists.

## ACKNOWLEDGMENTS

This study is based partly on data provided by the Association of American Medical Colleges (AAMC). The views expressed herein are those of the authors and do not necessarily reflect positions or policies of the AAMC. We thank Ericka P. von Kaeppler and Derek F. Amanatullah (Stanford University) for their assistance with data curation and Michael J. Brenner (University of Michigan) for helpful discussions.

## AUTHOR CONTRIBUTIONS

S.R.L. and V.B.M. had full access to all the data in the study and took responsibility for the integrity of the data and the accuracy of the data analysis. Drafting of the manuscript and statistical analysis: G.V. Critical revision of the manuscript for important intellectual content: S.R.L., R.J.W., and V.B.M. Administrative, technical, and material support and study supervision: S.R.L. and V.B.M.

## Supplementary Material
