# Supplementary material for: Trends in Specialty Training and National Institutes of Health Funding Among Surgeon-Scientists
Source: Ann Surg Open. 2024 Nov 25;5(4):e521. doi: 10.1097/AS9.0000000000000521 (PMC11661730; doi:10.1097/AS9.0000000000000521)
Supplement: Supplementary file 1 [file as9-5-e521-s001.pdf]

Supplemental Information

Table of Contents

Supplemental Table 1. K-awards by institution

Supplemental Table 2. Surgical subspecialty by degree type

Supplemental Table 1. K-awards by institution

	**Total K-Awards**
**INSTITUTION**	n = 476 (n%)
UNIVERSITY OF MICHIGAN AT ANN ARBOR	40 (8.4%)
JOHNS HOPKINS UNIVERSITY	37 (7.8%)
UNIVERSITY OF CALIFORNIA, SAN FRANCISCO	27 (5.7%)
DUKE UNIVERSITY	25 (5.3%)
STANFORD UNIVERSITY	18 (3.8%)
WASHINGTON UNIVERSITY	16 (3.4%)
UNIVERSITY OF PENNSYLVANIA	14 (2.9%)
NORTHWESTERN UNIVERSITY AT CHICAGO	13 (2.7%)
UNIVERSITY OF ALABAMA AT BIRMINGHAM	13 (2.7%)
UNIVERSITY OF CALIFORNIA LOS ANGELES	13 (2.7%)
UNIVERSITY OF PITTSBURGH AT PITTSBURGH	11 (2.3%)
UNIVERSITY OF SOUTHERN CALIFORNIA	11 (2.3%)
UNIVERSITY OF UTAH	10 (2.1%)
UNIVERSITY OF WASHINGTON	10 (2.1%)
OREGON HEALTH & SCIENCE UNIVERSITY	9 (1.9%)
UNIVERSITY OF TX MD ANDERSON CAN CTR	9 (1.9%)
INDIANA UNIV-PURDUE UNIV AT INDIANAPOLIS	8 (1.7%)
UNIV OF NORTH CAROLINA CHAPEL HILL	8 (1.7%)
UNIVERSITY OF CHICAGO	8 (1.7%)
UNIVERSITY OF ILLINOIS AT CHICAGO	8 (1.7%)
COLUMBIA UNIVERSITY HEALTH SCIENCES	7 (1.5%)
EMORY UNIVERSITY	7 (1.5%)
UNIVERSITY OF CALIFORNIA, SAN DIEGO	7 (1.5%)
UNIVERSITY OF IOWA	7 (1.5%)
UNIVERSITY OF WISCONSIN-MADISON	7 (1.5%)
MEDICAL UNIVERSITY OF SOUTH CAROLINA	6 (1.3%)
UNIVERSITY OF CALIFORNIA AT DAVIS	6 (1.3%)
UNIVERSITY OF FLORIDA	6 (1.3%)
UNIVERSITY OF ROCHESTER	6 (1.3%)
UNIVERSITY OF TEXAS MED BR GALVESTON	6 (1.3%)
UNIVERSITY OF VIRGINIA	6 (1.3%)
BAYLOR COLLEGE OF MEDICINE	5 (1.1%)

VANDERBILT UNIVERSITY	5 (1.1%)
YALE UNIVERSITY	5 (1.1%)
MEDICAL COLLEGE OF WISCONSIN	4 (0.8%)
NEW YORK UNIVERSITY SCHOOL OF MEDICINE	4 (0.8%)
OHIO STATE UNIVERSITY	4 (0.8%)
UNIVERSITY OF CINCINNATI	4 (0.8%)
UNIVERSITY OF COLORADO DENVER	4 (0.8%)
UNIVERSITY OF KENTUCKY	4 (0.8%)
UNIVERSITY OF MARYLAND BALTIMORE	4 (0.8%)
UNIVERSITY OF MIAMI SCHOOL OF MEDICINE	4 (0.8%)
UNIVERSITY OF MINNESOTA	4 (0.8%)
UNIVERSITY OF TEXAS HLTH SCI CTR HOUSTON	4 (0.8%)
WAKE FOREST UNIVERSITY HEALTH SCIENCES	4 (0.8%)
CLEVELAND CLINIC LERNER COM-CWRU	3 (0.6%)
UNIVERSITY OF ARIZONA	3 (0.6%)
DARTMOUTH COLLEGE	2 (0.4%)
GEORGE WASHINGTON UNIVERSITY	2 (0.4%)
ICAHN SCHOOL OF MEDICINE AT MOUNT SINAI	2 (0.4%)
PENNSYLVANIA STATE UNIV HERSHEY MED CTR	2 (0.4%)
UNIV OF MASSACHUSETTS MED SCH WORCESTER	2 (0.4%)
UNIVERSITY OF TENNESSEE HEALTH SCI CTR	2 (0.4%)
UT SOUTHWESTERN MEDICAL CENTER	2 (0.4%)
WEILL MEDICAL COLL OF CORNELL UNIV	2 (0.4%)
WEST VIRGINIA UNIVERSITY	2 (0.4%)
ALBERT EINSTEIN COLLEGE OF MEDICINE	1 (0.2%)
AUGUSTA UNIVERSITY	1 (0.2%)
CASE WESTERN RESERVE UNIVERSITY	1 (0.2%)
STATE UNIVERSITY OF NEW YORK AT BUFFALO	1 (0.2%)
THOMAS JEFFERSON UNIVERSITY	1 (0.2%)
UNIV OF MED/DENT OF NJ-NJ MEDICAL SCHOOL	1 (0.2%)
UNIVERSITY OF CONNECTICUT SCH OF MED/DNT	1 (0.2%)
UNIVERSITY OF LOUISVILLE	1 (0.2%)
UNIVERSITY OF MISSISSIPPI MED CTR	1 (0.2%)
UNIVERSITY OF NEBRASKA MEDICAL CENTER	1 (0.2%)
UNIVERSITY OF SOUTH ALABAMA	1 (0.2%)

UNIVERSITY OF VERMONT & ST AGRIC COLLEGE	1 (0.2%)
UPSTATE MEDICAL UNIVERSITY	1 (0.2%)
VIRGINIA COMMONWEALTH UNIVERSITY	1 (0.2%)

Supplemental Table 2. Surgical subspecialty by degree type

	**Total**	**MD**	**MD, PhD**	**MD + Other**
**Subspecialty**	n = 476 (n%)	n = 217 (n%)	n = 139 (n%)	n = 120 (n%)
Ophthalmology	113 (23.7)	33 (15.2)	56 (40.3)	24 (20.0)
Obstetrics & Gynecology	57 (11.9)	25 11.5)	3 (2.2)	29 (24.2)
Otolaryngology	56 (11.8)	25 (11.5)	19 (13.7)	12 (10.0)
Neurosurgery	34 (7.1)	20 (9.2)	11 (7.9)	3 (2.5)
Urology	33 (6.9)	18 (8.3)	7 (5.0)	8 (6.7)
Trauma/Critical Care	26 (5.5)	12 (5.5)	7 (5.0)	7 (5.8)
Surgical Oncology	23 (4.8)	13 (6.0)	3 (2.2)	7 (5.8)
Orthopedic Surgery	23 (4.8)	14 (6.5)	5 (3.6)	4 (3.3)
Transplant	22 (4.6)	7 (3.2)	10 (7.2)	5 (4.2)
Vascular Surgery	21 (4.4)	17 (7.8)	1 (0.7)	3 (2.5)
Cardiothoracic	21 (4.4)	7 (3.2)	3 (2.2)	11 (9.2)
Pediatric Surgery	13 (2.7)	8 (3.7)	4 (2.9)	1 (0.8)
Plastic Surgery	12 (2.5)	6 (2.8)	4 (2.9)	2 (1.7)
Colorectal Surgery	10 (2.1)	6 (2.8)	2 (1.4)	2 (1.7)
Bariatric Surgery	4 (0.8)	2 (0.9)	2 (1.4)	0 (0.0)
Endocrine	4 (0.8)	0 (0.0)	1 (0.7)	3 (2.5)
Breast	3 (0.6)	1 (0.5)	1 (0.7)	1 (0.8)
General	1 (0.2)	0 (0.4)	0 (0.0)	1 (0.8)
